# Bedside counseling on complementary medicine as a new model in pediatric cancer care—protocol of the SiKOM multicenter prospective cohort study

**DOI:** 10.3389/fped.2025.1567053

**Published:** 2025-04-14

**Authors:** Lara Nowak, Daniela Reis, Rebecca Büttner, Melanie Schwermer, Jennifer Bals, David D. Martin, Thomas Ostermann, Alfred Längler, Heidemarie Haller, Tycho Zuzak

**Affiliations:** ^1^ Department of Pediatrics, Gemeinschaftskrankenhaus, Herdecke, Germany; ^2^ Faculty of Health, Universität Witten/Herdecke, Herdecke, Germany; ^3^ Pediatric Hospital, Universitätsklinikum Tübingen, Tübingen, Germany; ^4^ Department of Psychology and Psychotherapy, Witten/Herdecke University, Witten, Germany; ^5^ Professorship for Integrative Pediatrics, Institute for Integrative Medicine, Witten/Herdecke University, Witten, Germany; ^6^ Center for Integrative Medicine and Planetary Health, University Hospital Essen, University of Duisburg-Essen, Essen, Germany; ^7^ Faculty of Medicine, University of Duisburg-Essen, Essen, Germany

**Keywords:** pediatric cancer, integrative cancer treatment, complementary medicine, safety, study protocol

## Abstract

**Introduction:**

An open dialogue between parents of children with cancer and medical staff about the benefits, risks, and interactions of complementary medicine used during cancer treatment is essential to enhance treatment safety and efficacy. However, both parents and medical staff often lack sufficient knowledge and willingness to engage in such discussions. To address this, bedside counseling for patients and families is proposed, provided directly by an external team comprising experienced specialists in complementary and conventional medicine. This approach aims to facilitate communication, improve understanding, and mitigate risks associated with complementary medicine during pediatric cancer care.

**Methods:**

This study will be conducted in five pediatric cancer centers in Germany. Physicians specializing in complementary medicine and pediatric oncology will provide bedside counseling to patients, parents, and local medical staff. Feasibility and change measures will be evaluated by comparing cohorts of parents who received complementary medicine counseling with those who did not, as well as medical staff before and after training sessions. Semi-structured interviews with parents and medical staff will further explore barriers to complementary medicine counseling and identify strategies to enhance its implementation. Quantitative data will be analyzed to assess the feasibility of the intervention, while qualitative data will provide in-depth insights into the perspectives and experiences of stakeholders.

**Results:**

The results will highlight predictors for effective, use-oriented counseling tailored to different target groups. This prototype support care model will serve as a framework for implementing complementary medicine counseling both within and beyond inpatient pediatric oncology settings. By integrating these findings, we aim to develop a replicable approach to complementary medicine counseling that addresses the unique needs of pediatric cancer patients, their families, and medical professionals.

**Discussion:**

Previous initiatives have primarily focused on training individual physicians within each center to address complementary medicine. In contrast, this novel strategy emphasizes direct bedside counseling for pediatric patients and families while providing ongoing support to local staff. This approach seeks to enhance treatment safety by reducing unintended interactions between complementary medicine and conventional cancer therapies, ultimately improving care quality and patient outcomes.

**Trial Registration:**

German Clinical Trials Register (DRKS), ID: DRKS00030478. Registered 22 December 2022 https://www.drks.de/DRKS00030478.

## 1 Introduction

The terms “Complementary Medicine” (CM) and “Alternative Medicine (AM)” are often used interchangeably but do in fact represent different concepts. Alternative treatments are used instead of conventional therapies, while complementary therapies are used in addition to conventional therapies when the latter are needed (1). Both therapy concepts encompass a broad spectrum of pharmacological and non-pharmacological, traditional and non-mainstream approaches, although for decades AM was mainly offered outside a conventional medical setting (2). This often causes unintended interactions with conventional treatments, in particular in cancer patients, and probably negatively impacts disease progression (3–5). Integrative medicine (IM) is an approach to medical care that recognizes the benefit of combining conventional (standard) therapies with complementary therapies that have been shown to be safe and effective. It uses the best evidence available in terms of efficacy and safety and focuses on the person as a whole (6).

In recent years, the use of CM has increased worldwide and now the World Health Organization describes CM as an important and often underestimated part of health care (7). There is growing interest and use of CM for both adults (8) and children (9, 10), as well as in adult and pediatric cancer patients (11, 12). Reasons given for the use of CM include side-effect management, physical stabilization and strengthening of the immune system, as well as the need to do something “extra” by oneself (11, 13, 14).

It is also worth noting that attitudes toward CM among health care professionals have become increasingly positive, with greater interest in and acceptance of integrating CM into conventional medicine (8, 15, 16). The use of CM in pediatric oncology requires the applicable IM concepts. However, as research shows, it is still unclear which CM treatment is used at which stage of conventional pediatric cancer treatment (11, 17). Längler et al. (11) assume that information about CM therapies is given to parents from a variety of sources such as relatives, friends, alternative practitioners, pharmacies and many others. In addition, online media are currently the most commonly used sources of information by parents with a pre-existing interest in alternative therapeutic options when seeking information about CM (17, 18). This information is often poorly comprehensible, often pertains to adults rather than to children, and can in some cases even be harmful due to misinformation or inappropriate content for young audiences (19). A major problem is that only about 50% of parents inform their pediatric oncologist about their CM use (10) and, equally, treating oncologists often do not actively ask about the CM use of their patients because of insufficient knowledge about CM (11, 14, 20, 21). In summary, CM counseling and support is a severely neglected area of physician-patient communication (22).

Parents of pediatric cancer patients emphasize the need for evidence-based information about effects and risks of CM, and healthcare professionals call for training opportunities on the safe use of CM to prevent unintended interactions with conventional therapies (18). In recent years, there have been promising concepts for improving knowledge about the safe use of complementary medicine (CM) in pediatric cancer centers, such as offering voluntary training courses to pediatric oncologists. These training programs have been provided by expert groups and professional organizations, aiming to equip oncologists with the necessary skills to address CM-related concerns (23). However, a limitation of these programs is that they predominantly involve physicians with a pre-existing interest in CM, which may introduce bias and reduce the generalizability of the findings. Specifically, pediatric oncologists without prior knowledge of CM are often insufficiently reached, although they could benefit the most from such training (24). Moreover, many cancer centers in Germany do not employ specialists with expertise in both CM and pediatric oncology (25).

From a broader perspective, several studies have examined the sources of knowledge and perceptions about CM among both parents and medical staff. Parents often rely on a mix of personal experiences, internet sources, and anecdotal reports from other parents, while medical staff generally acquire their knowledge through specialized training programs, clinical experiences, and guidelines, such as the German AWMF guidelines on CM in oncology (26). Despite the availability of such resources, there remains a significant gap in the practical implementation of CM counseling in pediatric cancer settings, where many parents still turn to external sources for guidance. The current project addresses this gap by involving an external team of pediatric cancer specialists consisting of the authors of this study providing counseling expertise in complementary and conventional medicine, providing counseling and training to medical staff and parents at five pediatric cancer centers in the Rhine-Ruhr region of Germany. This collaborative, interdisciplinary approach ensures that families receive evidence-based CM advice directly from trained healthcare professionals, mitigating the risks associated with self-researched information and enhancing the overall safety of pediatric cancer care (23, 24).

## 2 Objectives

The main objective of the study is to explore the feasibility and acceptability of implementing CM counseling and supporting strategies in conventional pediatric cancer centers without in-house specialized CM counseling services. Feasibility refers to the extent to which a new intervention, program, or procedure can be successfully delivered in a specific context that is not fully controlled. It involves assessing whether the implementation processes can be carried out as intended in real-world settings, considering various logistical, operational, and contextual factors (Berry & Shabana 2020). Throughout this manuscript, the term “medical staff” is used comprehensively to refer to a diverse group of professionals involved in the treatment and care of children. This includes not only physicians but also nurses, therapists, and social educators. The broad application of this term reflects the interdisciplinary nature of pediatric care, in which various professional groups collaborate to ensure holistic and effective treatment. Beside training of medical staff in the dos and don'ts of CM, personal counseling of parents is taking place directly at the patients' bedside about how they handle CM used in parallel with conventional treatment, what kind of CM is allowed, what they should omit and what is dangerous.

Acceptability, on the other hand, pertains to the extent to which the target population and stakeholders involved in the implementation perceive the intervention or procedure to be satisfactory, appropriate, and agreeable. This includes their comfort with, perceptions of, and reactions to the intervention, which can significantly influence its successful uptake and sustainability [Berry & Shabana, 2020; (27)]. This intervention will be scientifically evaluated in relation to the following acceptability criteria:.

1. What are the needs of parents of children with cancer and medical staff regarding counseling on the safe use of CM?
2. Is the CM intervention that has been developed acceptable for the parents of children with cancer as well as for medical staff?

The following feasibility issues are also central to the study:

1. Is it feasible to recruit a sufficient number of parents/medical staff to obtain representative cohorts?
2. Is it feasible to implement CM interventions in different conventional study centers according to the protocol or are there barriers that will require protocol adjustments?

Secondary objectives include exploratory outcomes for evaluation:

1. Do trained parents feel better informed about the risks, benefits and safety of CM compared to untrained parents, and does this increase their ability to make better informed decisions about their use of CM?
2. Do attitudes towards CM and CM usage differ between trained and untrained parents?
3. To what extent does the medical staff feel relieved from administrative tasks, time pressure, and professional uncertainty by the external provision of CM counseling for parents?
4. Can knowledge of the benefits and risks of CM among medical staff and their attitudes towards it be improved?

The results should make it possible to successfully implement and establish CM counseling and supporting strategies in conventional pediatric cancer centers. In addition, individual CM counseling concepts will be developed for other clinics in the long term, in order to meet evidence-based IM recommendations (6).

## 3 Methods and analysis

The study protocol was drawn up according to the standard protocol items for clinical trials (SPIRIT) guideline (28) as well as Consolidated Standards of Reporting Trials (CONSORT) (29). Each study site was approved by the respective ethics committee and registered at the WHO International Clinical Trials Registry Platform/German Clinical Trials Register (DRKS00030478) before patient recruitment. The inclusion of patients and healthcare providers is essential in our study to integrate their perspectives into the research process. Their active involvement in developing survey instruments and pilot-testing study procedures ensured relevance and user-friendliness. Additionally, continuous feedback through a dedicated hotline and suggestion boxes allows for real-time adjustments and improvements to the study experience.

### 3.1 Study setting and design

Several large centers for pediatric oncology and hematology are located in the Rhine-Ruhr area, with more than 30% of all pediatric cancer patients in Germany being treated in this region. The Gemeinschaftskrankenhaus Herdecke, Germany, is a pediatric oncology center according to the quality criteria of the Federal Joint Committee (Gemeinsamer Bundesausschuss, G-BA), a member of the Society for Pediatric Oncology and Hematology-network (Gesellschaft für Pädiatrische Onkologie und Hämatologie, GPOH) and the coordinating study center. Further participating centers are:

- Vestische Children's and Youth Hospital Datteln, Germany
- Pediatric Hematology/Oncology, Dortmund Hospital, Germany
- Department of Pediatrics and Adolescent Medicine, University Hospital Essen, Germany
- Pediatric Hematology/Oncology, University Hospital Cologne, Germany

**Figure 1:**
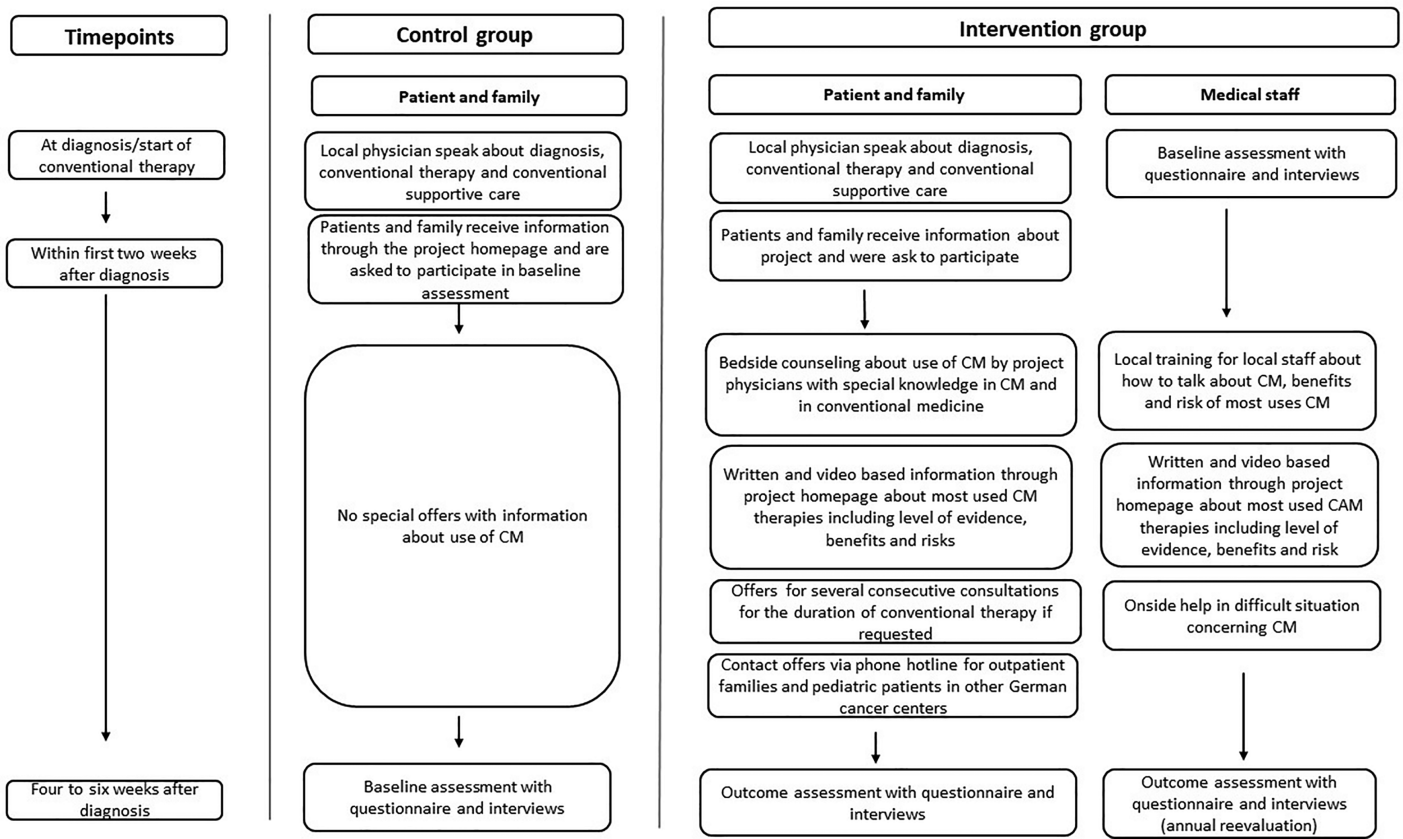
Study flow chart with control and intervention group and an overview of processes that will run consecutively in each participating cancer centers individually.

The Rhine-Ruhr metropolitan area is beneficial for the fact that there are many oncological centers in a very small area, so that the treatment team can drive to them and deliver bedside counseling and support in presence. By selecting a prospective, multicenter study design, two cohorts of parents of children with a diagnosis of cancer and one cohort of medical staff from the participating pediatric oncology departments can be included. Sociodemographic data will be collected from all cohorts, with particular emphasis on the educational level of the parents, to determine whether education influences responses in the surveys. While one parent cohort will receive CM counseling, the other will serve as a waitlist cohort to be offered the respective CM counseling after the outcome assessment. Group assignment will be non-randomized by starting with a control cohort followed by the intervention cohort. After being informed about the study during the initial visit by their treating physician and signing written informed consent, parent cohorts will receive standard questionnaires three weeks after study inclusion. For the parent control cohort, the CM counseling will take place after this outcome assessment, while the parent intervention cohort will receive CM counseling before. Outcomes will be compared between groups. Medical staff will be trained in CM counseling strategies and assessed before and after the CM training. Outcomes will be compared within this group. A summary of the study design can be found in Figure 1. The active study period of recruiting is scheduled from December 2022 to end of 2027. Since there are five participating centers and a small project team, the time invested to be able to include subjects in the study will be in relation to the effort involved. For the medical staff, 100 participants are considered an acceptable and desirable number. Traveling to the study centers and maintaining contact with parents requires resources. The sample size for the survey and consultation includes 50 participants, evenly divided into two cohorts of 25 individuals each. We also expect a dropout of 10%, as parents who find themselves in such a difficult situation might also turn away.

### 3.2 Eligibility criteria

To be included in the study, participating parents must have reached the age of 18. Their children should be under 18 years of age. Both parents have to give written informed consent to participate in the study. Children who have already reached the age of 16 must give their written consent as well. All first-onset oncological diseases are considered eligible diagnoses and the children have to be undergoing conventional cancer treatment in one of the participating cancer centers. Parents who already have experience in dealing with CM can still be part of the study.

Medical staff will be eligible if they are employed in one of the participating study centers within the framework of pediatric oncology and provide written informed consent. Insufficient knowledge of German is considered an exclusion criterion for both parents and medical staff.

### 3.3 Selection and treatment of subjects

Parents and their children who meet the inclusion criteria are recruited at the participating study centers and made aware of the study during the initial visit with their treating on-site oncologist. Afterwards, enrolled pediatric patients and their parents will be informed by study staff about the nature, significance and scope of the study. They will also receive written confirmation that no disadvantages in medical treatment of their children will occur if they do not participate.

The medical staff will also be recruited at the participating study centers by the study team, who will inform pediatric oncologists and nurses about the study and hand out written study information. The collection of data will take place on site. If participants can no longer be reached on site, further data collection will be carried out by mail or online.

### 3.4 Interventional methods

The intervention for parents will include a half-hour CM counseling session at the respective treatment center and, if necessary, an ongoing exchange. The personal counseling and support are planned to take place directly at the bedside and includes advice on how to safely use CM therapies in addition to conventional cancer therapy. In this setting, parents have the opportunity to ask questions of interest about CM. To ensure comprehensive reporting and implementation of the intervention, we will utilize the Template for Intervention Description and Replication (TiDier) framework (30). This framework will guide the detailed description of the intervention components, delivery methods, and the context in which it is implemented. Additionally, a fidelity checklist, informed by the GRIPP2 reporting framework, will be employed to assess the consistency and accuracy in delivering the intervention across different sites and facilitators (31).

The intervention for the medical staff will consist of frontal training units on the communication of CM topics mainly related to the effects, adverse events and interactions of different CM therapies with conventional cancer treatment. This is supplemented by case-based training in each of the five participating centers.

For the parent intervention cohort as well as the medical staff cohort, evidence-based information is made available on the SiKOM homepage (www.sikom.info) and by means of brochures for all those interested in further CM information. Moreover, study participants have the opportunity to ask questions or receive information on individual concerns via a telephone hotline, which is available throughout the entire duration of the project.

## 4 Outcomes

**Figure 2:**
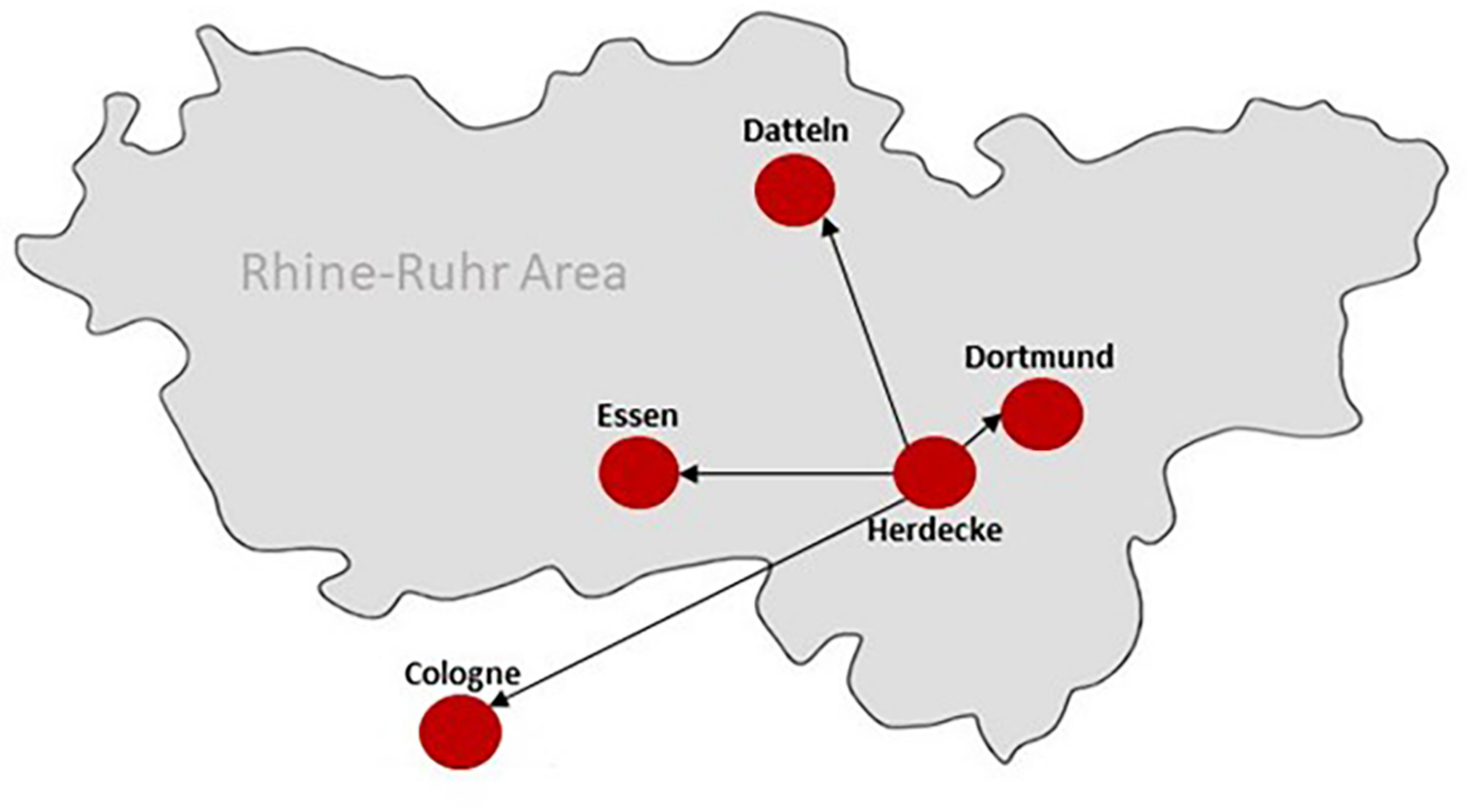
Overview of the study centers in the rhine-ruhr area in Germany. The team in Herdecke, consisting of medical and psychological staff with expertise in CM as well as in pediatric oncology offers bedside counseling for patients and parents and support for on-site medical staff in all five participating pediatric cancer centers.

All participants will be asked about baseline social demographics. Data on pediatric cancer characteristic will be obtained from medical records. Both cohorts of parents receive one questionnaire each, while medical staff receive questionnaires twice, once before and once after the CM training (see Figure 2). The questionnaires refer to the objectives already mentioned above in the protocol.

### 4.1 Feasibility

1. Attitudes towards [ABCAM (32)], previous experience with [I-CAM-R (33, 34)] and support needs (LS (35) regarding CM of parents of children with cancer
2. Number of parents who can be screened, enrolled and counseled during the study period, number of medical staff, study-related retention and dropout rates, reasons for study dropouts
3. Acceptability of the CM intervention (parent intervention cohort only), assessed with the Acceptability of Intervention Measure [AIM (36)]
4. Proportion of planned consultations that could be completed, feasibility of the intervention by medical staff [FIM (36)]
5. Acceptability of the overall approach within the project is assessed in a targeted sample of parents and medical staff [TFA (37)].

### 4.2 Evaluation

1. Parents satisfaction with the provision of information on the use and safety of CM and their ability to make informed decisions about the use of CM [PS-CATE (38)], [SDM (39)]
2. Change in attitudes towards CM, assessed with the Attitudes and Beliefs about Complementary and Alternative Medicine [ABCAM (32)] and the benefits of potentially harmful CM procedures [I-CAM-R (33, 34)]
3. Perceived stress of parents, assessed by the Perceived Stress Scale [PSS (40)], and anxiety and depression, assessed by the Patient Health Questionnaire [GAD/PHG (41)], training needs, and content and structural relief of medical staff [LS (35)]
4. Attitudes towards CM procedures, assessed by the Complementary and Alternative Medicine Health Belief Questionnaire [CHBQ (42)], knowledge, query, application, reasons for non-application of CM in everyday clinical practice [LS (35)]

### 4.3 Primary outcome

These outcome measures are considered in the overall study concept. Using the questionnaires, we examine changes in the above measures of feasibility; and we evaluate the project by interviewing the two cohorts of parents and medical staff before and after the training. The list of these measurement points should not be seen as a ranking, but rather elaborates the hypotheses to be tested: Firstly, whether the project has an influence on parental satisfaction with the information on CM. Secondly, its influence on the ability to make decisions. Thirdly, its influence on attitudes towards CM. Lastly, its influence on perceptions of personal stress levels. For the feasibility of the project, special attention is paid to practicability. The focus is on the different processes that take place at different points in time. Both parents and medical staff as well as the project team should accept the procedures in order for the project to be considered feasible. The completed questionnaires and the qualitative interviews are used to assess acceptance. Furthermore, the number of participants in the questionnaires as well as in the subsequent consultations show whether the project is future-oriented. Minor adjustments should also be feasible afterwards, if relevant for the overall outcome.

### 4.4 Secondary outcome

In addition to quantitative data, qualitative data will be assessed using semi-structured interviews of the parent intervention cohort and medical staff. In the *a priori* categorization, gender and age characteristics are taken into account and, in the case of medical staff, also their job description or position in the organization. To ensure the interviewees’ initiative was respected, each interview was limited to 20 min, with a primary focus on identifying barriers to implementation. The pre-developed interview guide shown in Table 1 is intended to identify barriers to the implementation of the CM intervention. Results of the qualitative analysis will be used to improve the CM training and counseling sessions, if necessary, as well as the questionnaire.

**Table 1:** Semi-structured interview guide for parent interviews and interviews with medical staff.

Overview	Content	Parents	Medical staff
Introduction	Welcome and data protection Introduction of the interviewer Procedure and aim of the interview		
Checking the quota criteria	Socio-demographic data of the interviewee	Household/family situation Field of diagnosis	Educational background Job description/field of activities State of knowledge of the SiKOM project
Introductory questions	Definition of CM Attitude towards CM Relevance of CM in (clinical) everyday life	Guiding question: What do you understand by CM? Maintenance question: What else counts as CM for you?	
		What role does CM play in your everyday life? To what extent do you use CM? Can you give an example? To what extent do you ask your doctor about CM? Have there already been conflicts in regard to CM and your child’s current therapy?	What role does CM play in your clinical life? To what extent do parents ask you about CM? To what extent are CM therapies used in your clinic? Can you give an example? Have there already been conflicts in regard to CM and the current therapy of your patients?
Main questions	Bedside CM counseling for families Group training and general information on the project	Guiding question: What should ideal counseling look like for you? Maintenance questions: What do you consider should definitely be part of CM counseling? What should be the central message? Where do you see possible barriers to the introduction of counseling?	Guiding question: What should ideal counseling look like for patients? Maintenance questions: What do you consider should definitely be part of CM counseling for patients? What should be the central message? Where do you see possible barriers to the introduction of counseling? Guiding question: What should training on CM look like for you? Maintenance questions: Which contents must not be missing within the training? Through which channels would you like to be trained? What are strengths of the project, and do you have any concerns about it?
Closing question	Additions Space for questions from the interviewee		
Conclusion	Acknowledgement Farewell		

### 4.5 Data analysis

Frequent communication between the project team and contact persons at the respective pediatric oncology departments and the parents, as well as the regular on-site monitoring of the study team, will ensure the correctness and quality of the data generated. All parent and staff questionnaires from the five centers are stored separately from the consent forms. The questionnaires to be evaluated are only available to the statistician in a pseudonymized form. Pseudonymization involves the removal of personal identifiers and their substitution with placeholder values, typically composed of a sequence of numbers and letters, so it is not possible to draw conclusions about the individual participants. The data analysis is carried out by IBM® SPSS. The questionnaires are entered manually into SPSS and checked for transmission errors using the dual control principle with the original data.

Qualitative data will be audio-recorded, transcribed verbatim, anonymized, and then coded and analyzed according to qualitative content analysis (43, 44) using MAXQDA software. The analysis follows an inductive approach, as the categories were independently developed based on our research question.

To evaluate the feasibility and determine whether the intervention is suitable for progression to a larger effectiveness trial, specific progression criteria have been established. Recruitment feasibility will be assessed by the ability to recruit the planned sample size of 100 medical staff and 50 parents within the study period, with an acceptable participation rate and minimal refusal. Retention rates are also critical, with at least 80% of participants expected to remain engaged throughout the study, and reasons for dropouts systematically documented. The acceptability of the intervention will be evaluated using the Acceptability of Intervention Measure (AIM) and qualitative feedback, with positive feedback from at least 70% of participants serving as a benchmark.

Additionally, compliance will be assessed by ensuring that at least 80% of planned counseling sessions are completed, while any barriers to implementation, such as logistical or organizational challenges, will be identified through qualitative interviews with staff. Data quality is another essential criterion, with at least 90% of collected questionnaires expected to be complete and analyzable to ensure the reliability of feasibility and outcome measures. Finally, satisfaction rates among parents and medical staff regarding counseling content and delivery will be monitored, and specific barriers to implementation will be identified through semi-structured interviews.

These criteria provide a structured basis for determining the feasibility of the intervention and whether it warrants further investigation in a fully powered randomized controlled trial (RCT). If certain criteria are not met, modifications may be made, and feasibility reassessed in subsequent phases.

### 4.6 Statistics

As this is an exploratory study, the sample size was not predetermined. However, a target of 100 participants for the cohort of medical staff, along with two groups of 25 participants each for the parent cohort has been set. The chosen sample sizes are justified based on feasibility considerations and the estimated response rates. Across the four study centers, an average of five new patients are admitted each month. All eligible patients and their parents who do not immediately decline participation will be included in the study. Based on prior experiences, it is estimated that approximately 50% of distributed questionnaires will be returned completed.

This corresponds to an expected return of around 2–3 questionnaires per month per center from the parents. With these projections, the parent cohort would reach the target size of 50 participants within approximately 6–8 months. For the cohort of medical staff, which is relatively stable in size, we anticipate achieving the target sample size within a similar period, assuming a participation rate of around 70%. A 95% confidence interval will be calculated for the proportion of participants who return completed questionnaires, allowing us to assess the precision of our estimates. This approach ensures that the sample size is sufficient to explore key outcomes while remaining realistic and aligned with the study's feasibility constraints.

The statistical analysis plan includes descriptive statistics for sample characteristics, feasibility, and effectiveness outcomes. Descriptive statistics comprise percentages for categorical variables and means with standard deviations for continuous variables. Feasibility results are analyzed by reporting the number and proportion of parents screened, enrolled, and counseled during the study period, along with retention and dropout rates. Reasons for dropouts are categorized and analyzed descriptively. Subgroup analyses explore variability across demographic groups, and qualitative interviews provide additional insights into perceived acceptability. For medical staff, feasibility is measured using the Feasibility of Intervention Measure (FIM), with descriptive statistics summarizing scores and qualitative interviews identifying barriers to implementation. Completion rates of planned counseling sessions are also reported descriptively, and differences across study centers are explored using chi-squared tests or Fisher's exact tests.

Effectiveness outcomes are analyzed by examining parental satisfaction with counseling, including clarity of information on complementary medicine (CM) and its safety, using the PS-CATE and SDM-Q-9 scales. Means and standard deviations are reported, and paired *t*-tests or Wilcoxon signed-rank tests are applied to compare satisfaction scores before and after the intervention. Changes in parents’ ability to make informed decisions are assessed through pre- and post-intervention scores on the SDM-Q-9. Changes in parental attitudes toward CM, as measured by the ABCAM, and perceptions of the benefits of potentially harmful CM procedures, as assessed with the I-CAM-R, are analyzed using paired t-tests for within-group comparisons. Between-group differences, such as those between intervention and baseline cohorts, are tested using independent t-tests or Mann–Whitney *U*-tests. For medical staff, training needs and perceived burden are assessed using Likert scales, while changes in knowledge, attitudes (CHBQ), and practices regarding CM are evaluated with paired *t*-tests. Subgroup analyses are performed to identify predictors of changes in these outcomes.

To ensure psychometric robustness, subscales of multi-faceted questionnaires are averaged into non-weighted indices by calculating the mean of individual items where applicable. Cronbach's alpha coefficients are computed to assess the internal consistency of scales. All statistical tests are exploratory and conducted at a significance level of alpha = 0.05, without adjustments for multiple testing. For significant changes, additional regression analyses are conducted to identify predictors of change in predefined outcomes such as satisfaction, attitudes, or decision-making ability. Qualitative data are analyzed using thematic content analysis to complement the quantitative findings. This comprehensive approach ensures a robust evaluation of the feasibility, acceptability, and effectiveness of the intervention while identifying potential areas for refinement.

## 5 Discussion

The diagnosis of cancer almost always represents a life-changing moment for the affected patients and their families, which is accompanied by a serious change in life circumstances and the subsequent search for different therapeutic options (45). In this context, questions about CM therapies are often raised in families with a child suffering from cancer. Previous projects are based on the fact that the parents had to travel long distances to consult reputable or dubious therapists and incur additional financial costs. The aim of this project is to establish professional bedside counseling and support for parents in their main hospital. Our main focus is to improve care of children with cancer by providing the best possible support regarding CM therapies. For this purpose, the current evidence level regarding the most-used CM therapies in pediatric oncology will be addressed and parents can obtain information through multiple channels. This project should also substantiate that parents are less stressed and have to make less effort to get the information they need. In addition, we would like to show that the team members treating the patient also benefit from professional support so that they are less frequently confronted with questions they do not know the answers to and have at expertise available on site from with to get support.

### 5.1 Strengths

The present study will assess the feasibility of integrating CM into pediatric oncology and should serve as a new model for considering CM in relation to conventional concepts. Further results of this prospective cohort study will provide information on the decision-making ability of parents as well as on the feasibility and barriers to CM counseling. At this point, it must be emphasized that the outcome measures of the questionnaires only provide a direction and must be considered in the overall concept. By carrying out all planned project steps, the practicability is tested at the same time as basic information for carrying out the study on a larger scale in the future is provided. In this way, possible future obstacles can be circumvented before implementation. In addition, analyses of staff data will highlight support needs and barriers related to CM training. The intention is that medical staff learn to apply CM methods more confidently and are able to respond individually to the needs of their patients or the patients’ parents in the future. In the future, training will enable pediatric oncologists to provide their cancer patients with evidence-based advice on the safe use of CM. This may bring about an improved standard of care by reducing side-effects of CM and improving adherence to cancer treatments.

Both patients and staff alike have to be supported directly in the respective center because families often cannot leave the hospital for several weeks and time-consuming trips to unexperienced therapists could be avoided. A particular challenge in this study is networking within the five participating centers. In order to establish and maintain personal contact, it is mandatory for the project members to regularly visit the individual study centers and maintain a close exchange of information, feedback and ideas. To address this challenge, adequate time for networking is planned, and continuous efforts are made to optimize communication. This involves focusing on the parents participating in the questionnaires and consultations, seeking positive feedback from the medical staff, and ultimately analyzing the questionnaires to determine if sufficient time was invested in exchanging information with other study centers.

### 5.2 Limitations

A further task is to ensure the compliance of the parents as well as the medical staff. Parents whose children have cancer face an emotional challenge and may find it difficult to engage with the trial. Studies show that an informed patient influences treatment-related expectations and patient engagement (46, 47). In addition, the parents are already burdened with substantial documentation and paperwork related to conventional studies, consent forms, and various other requirements. For this reason, solid communication skills and authentic empathy are required on the part of project staff (48), and the project team has been specifically trained to meet these demands. At the same time, hospital staff have many patients and therefore little time for additional activities. Compliance and commitment of both groups is maintained through close, personal contact in the form of educational talks as well as the possibility of telephone feedback. In addition, counseling and support will take place on site at the respective facility and strengthen engagement.

A possible effect could be that participants tend to respond in a socially desirable way, knowing that the study deals with attitudes and acceptance towards CM. To minimize this bias, we will ensure anonymity, stress the absence of right or wrong answers, ask neutral questions, and include implicit honesty prompts. Additionally, indirect questioning techniques will be used where appropriate to reduce socially desirable responses. These measures aim to encourage participants to disclose their true opinions and behaviors. Furthermore, a generalization of the results of the evaluation to Germany as a whole may be limited, as five out of 62 study centers in Germany will participate (8%). With regard to the generalizability of the results of the evaluation, it should be mentioned that individuals who are interested in CM may be more likely than other to participate in the survey. However, it could also be useful for people who have a rather positive attitude towards CM to be informed about its safe use. The need to improve communication about CM in pediatric oncology is also underlined by other authors (48).
